# Solute Diffusion into Polymer Swollen by Supercritical CO_2_ by High-Pressure Electron Paramagnetic Resonance Spectroscopy and Chromatography

**DOI:** 10.3390/polym13183059

**Published:** 2021-09-10

**Authors:** Oleg I. Gromov, Mikhail O. Kostenko, Alexander V. Petrunin, Anastasia A. Popova, Olga O. Parenago, Nikita V. Minaev, Elena N. Golubeva, Mikhail Ya. Melnikov

**Affiliations:** 1 Faculty of Chemistry, Lomonosov Moscow State University, Leninskiye Gory, 1-3, 119991 Moscow, Russia; nas.popova.04@gmail.com (A.A.P.); oparenago@scf-tp.ru (O.O.P.); legol@mail.ru (E.N.G.); melnikov46@mail.ru (M.Y.M.); 2 Kurnakov Institute of General and Inorganic Chemistry of the Russian Academy of Sciences, Leninsky Prosp. 31, 119991 Moscow, Russia; kostenko@supercritical.ru; 3 Institute of Physical Chemistry, RWTH Aachen University, 52056 Aachen, Germany; alex.petrunin.29@gmail.com; 4 Federal Scientific Research Centre “Crystallography and Photonics” RAS, Institute of Photon Technologies, Pionerskaya Str. 2, Troitsk, 108840 Moscow, Russia; minaevn@gmail.com

**Keywords:** poly(D,L-lactide), supercritical fluid, carbon dioxide, diffusion, electron paramagnetic resonance

## Abstract

High-pressure electron paramagnetic resonance (EPR) was used to measure translational diffusion coefficients (D_tr_) of a TEMPONE spin probe in poly(D,L-lactide) (PDLLA) and swollen in supercritical CO_2_. D_tr_ was measured on two scales: macroscopic scale (>1 μm), by measuring spin probe uptake by the sample; and microscopic scale (<10 nm), by using concentration-dependent spectrum broadening. Both methods yield similar translational diffusion coefficients (in the range 5–10 × 10^−12^ m^2^/s at 40–60 °C and 8–10 MPa). Swollen PDLLA was found to be homogeneous on the nanometer scale, although the TEMPONE spin probe in the polymer exhibited higher rotational mobility (τ_corr_ = 6 × 10^−11^ s) than expected, based on its D_tr_. To measure distribution coefficients of the solute between the swollen polymer and the supercritical medium, supercritical chromatography with sampling directly from the high-pressure vessel was used. A distinct difference between powder and bulk polymer samples was only observed at the start of the impregnation process.

## 1. Introduction

Since pioneering reports by Berens [1] and Sand [2], the impregnation of polymers using supercritical fluids (SCFs) has remained of great interest in many applications, such as the loading of biocompatible materials with drugs [3,4,5] and food packaging materials with antioxidants, antimicrobials, etc. [6,7]; supercritical polymer dyeing [8,9,10,11,12,13]; fabrication of nanocomposites [14,15,16,17]; and polymer blends [18,19,20], just to name a few. SCFs make these approaches to polymer modification efficient and environmentally friendly. One of the most “green” supercritical media that is compatible with biomedical and food applications is carbon dioxide. It is broadly used due to its remarkable miscibility with a large number of polymers [21] and a high solvating capacity in the supercritical state. Supercritical CO_2_ (scCO_2_) has been shown to plasticize polymers by increasing their fractional free volume and decreasing intermolecular interaction energy [22]. Due to this effect, the diffusion of additives into the polymer bulk proceeds faster [1,12], and leads to their uniform distribution in a matrix [23,24].

For technological applications, it is important to predict the amount of an impregnated substance and its distribution in a polymer. Therefore, a solute diffusion rate in the swollen polymer matrix and its distribution coefficient between the SCF medium and the polymer are needed for this to be known. Under normal conditions, the diffusion rate of a solute in a medium such as solvent-swollen polymers or porous materials soaked with a solvent is measured using different methods, such as the diffusion (diaphragm) cell and infinite cylinder/semi-infinite slab methods (with either uptake measurement or sectioning/imaging to obtain the concentration profile) [25], electrochemical methods [26], NMR methods (Pulsed Field Gradient NMR, NMR imaging, etc.) [27,28], EPR methods (concentration broadening measurement, EPR imaging) [29,30], IR and Raman spectroscopy/microscopy [31,32,33,34], FRAP technique [25], forced Rayleigh scattering [35,36], and so on. All these methods have advantages and disadvantages in terms of required time and effort, sample opacity, need for special molecular probes, cost, and availability of the equipment. The use of a supercritical medium as a solvent further complicates these measurements: either specialized equipment is needed to carry in situ measurements under high pressure, or the sample should be removed from the supercritical medium to carry ex situ measurements under ambient conditions. Most often, the amount and distribution of the solute are evaluated ex situ by conventional spectroscopic methods [12,23,31,37] or gravimetrically [38]. However, those ex situ methods are quite laborious as every experimental point needs its distinct supercritical experiment, while the depressurization step, which precedes such measurements, can dramatically influence the properties of the resulting material [39] and contribute to the removal of the solute from polymer due to the convective mechanism [40,41]. Therefore, to save time and materials, it is desirable to follow the impregnation process directly under supercritical conditions.

In situ infrared spectroscopy in transmission [4], or ATR mode [42], has been widely used to study the impregnation kinetics and probe the molecular state of the impregnated substance (dispersed vs aggregated, hydrogen bonding to the polymer) [42,43,44]. In addition to IR spectroscopy, in situ UV/Vis spectroscopy has been proposed to characterize polymer impregnation with dyes [11,45]. Raman spectroscopy has been used to study the diffusion of ethanol in aerogels during supercritical drying [34], and is also able to characterize the state of the solute resolved in space. High-pressure NMR imaging and forced Rayleigh scattering were also used to measure tracer diffusivities in CO_2_ plasticized polymers [28,46].

Electron paramagnetic resonance (EPR) spectroscopy is similar to nuclear magnetic resonance (NMR) spectroscopy, but detects compounds containing unpaired electrons such as radicals or transition metal complexes. Most substances are EPR-silent; hence, the EPR, or rather spin-probe EPR, approach is suitable and frequently used to evaluate the microstructure and dynamics of polymers [47] by tracking the paramagnetic probe inside the matrix of interest. Surprisingly, EPR has never been used to evaluate the transport properties of swollen polymers and porous materials in SCFs. Moreover, EPR has hardly ever been used for studying polymer/SCF systems [48,49]. The spin-probe EPR approach tracks paramagnetic probe molecules, which are usually stable nitroxide radicals or transition metal complexes. Even though the need for spin probes can be considered a significant drawback, on the other hand EPR is extremely sensitive to the microenvironment and dynamics of the radical technique and most materials are completely transparent to it, while quantitative radical amount measurements are achieved through spectrum double integration. EPR makes it possible to evaluate the mobility of a probe on multiple spatial scales [50]: rotational mobility is readily obtained from the shape of the EPR spectrum, molecular-scale translational mobility (<10 nm path) can be obtained by exchange interaction rate measurement, and, finally, EPR imaging and probe uptake measurement deliver macroscopic diffusivity. High-pressure/in situ EPR spectroscopy uses equipment similar to that of high-pressure NMR [51], and has been used so far to evaluate density enhancement and exchange reactions in SCFs [52,53,54,55,56,57], clustering of the solute in SCFs [58,59,60,61,62], microemulsion formation [63,64], and glass transition pressure in polylactide and polylactide-co-glycolide subjected to subcritical CO_2_ [48]. In addition, it has been reported that the mobility of spin probes can reflect the distribution of free volume in polymers plasticized by subcritical CO_2_, similar to a conventional plasticizer [49].

Commonly used nitroxide spin-probes are soluble to a necessary extent in SCFs such as scCO_2_ [54], and easily penetrate many polymers from SCFs [24,65,66,67,68]. This makes it possible to study the transport properties of polymers swollen in SCFs using high-pressure EPR, while, to the best of our knowledge, this method has been used so far to characterize systems in equilibrium only. We believe that this approach can be particularly useful for studying the kinetics of impregnation: the amount of impregnated spin probe, its local concentration, and the microstructure of its surroundings can be tracked simultaneously.

Obtaining the distribution coefficients of a solute between the supercritical medium and swollen or soaked phase is a crucial step to employ in situ diffusivity measurements. Unfortunately, the EPR technique is not particularly well suited for this purpose due to the sample geometry. Indeed, a common EPR tube requires a narrow and relatively long sample, which is quite unfavorable in terms of the time required to achieve equilibrium in an in situ experiment, while the drawbacks of the ex situ EPR measurements after a continuous impregnation are obvious. For that reason, we adopted a method to quantitatively analyze the concentration of a substance dissolved in the supercritical phase directly, using supercritical chromatography. The minimization of the sampling effect on equilibrium is usually achieved by eliminating dead zones, extracting the minimum amount of the medium using multiport valves with dosing loops [69], and increasing the volume of the autoclave, which also allows the use of more favorable polymer sample geometries, e.g., larger surfaces and smaller diffusion paths. A supercritical medium sample is transferred into a form that is suitable for analysis, for example, into a solution in a liquid solvent for subsequent spectrophotometry [70] or, as shown in [71], the analysis is carried out without depressurizing the sample directly in the form of a supercritical fluid with the application of supercritical fluid chromatography. The latter option is especially convenient due to the absence of an additional stage of sample preparation, which reduces time costs and the probability of error introduction. In addition, direct quantitative analysis of the supercritical phase using UV and IR transmission spectrometry, using high-pressure cells with optically transparent windows or optic fibers, is possible [72,73,74]. Supercritical chromatography still has an advantage over these approaches, and the in situ gravimetric approach, as it allows for the simultaneous measurement of different compound concentrations in a single experiment.

Herein we apply supercritical chromatography to obtain a distribution coefficient, and in situ EPR spectroscopy to study the kinetics of supercritical fluid impregnation of a poly(D,L-lactide) swollen in supercritical CO_2_. Poly(D,L-lactide) was chosen as a model system because polylactides and their co-polymers constitute an important class of biodegradable polymers for tissue engineering and drug delivery that can be impregnated with desired additives using SCFs [75,76,77,78]. We propose a possible mechanism by analyzing the kinetic data in combination with the molecular motion and local concentration of a nitroxide spin probe.

## 2. Materials and Methods

### 2.1. Materials

Nitroxide radical TEMPONE (4-oxo-2,2,6,6-tetramethylpiperidine-N-oxyl; 97% purity, Sigma Aldrich) was used as received. Poly(D,L-lactide) PURASORB^®^ PDL04 (Purac Biochem, Gorinchem, Netherlands) with inherent viscosity 0.4 g/dL and T_g_ = 56 °C was mechanically powdered. Chemically pure carbon dioxide (99.998% grade, NIIKM Ltd., Moscow, Russia), acetone (>98%, Moscow, Chimmed, Russia), ethanol (~95%, Moscow, Ferein), and methanol (HPLC gradient grade, Moscow, Chimmed, Russia) were used as received.

### 2.2. Measurement of Equilibrium Distribution between Polymer and scCO_2_ Using Supercritical Chromatography

**Figure 1:**
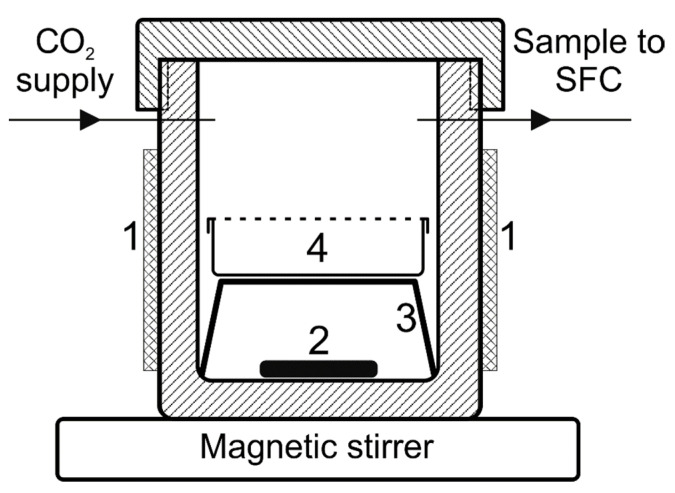
Schematic representation of autoclave interior: 1—heating jacket, 2—magnetic stirrer bar, 3—vial support, 4—glass vial with perforated cap for polymer sample.

The distribution coefficient of TEMPONE between the scCO_2_ and polymer measurement was carried out using a custom-built system, described in [79], which included a high-pressure autoclave (stainless steel, total internal volume 150 mL, effective volume 143 mL, Figure 1), a sampling device (6-port valve Valco C6W equipped with a 10 µL dosing loop, VICI AG International, Schenkon, Switzerland), and a supercritical fluid chromatograph (Acquity UPC2 with a diode array spectrophotometric detector, Waters, Milford, MA, USA). General principles are described in [80,81].

A weighted amount of TEMPONE (typically 10–15 mg) and a vial with a PDLLA sample (typically ~100 mg) were placed in the autoclave. The autoclave was then sealed, heated, and filled (Supercritical-24 pump, Teledyne-SSI, State College, PA, USA) with CO_2_ to the desired temperature and pressure. Under the considered conditions, swollen PDLLA liquefied and formed a thin film at the bottom of the vial, leading to relatively fast equilibration of TEMPONE concentrations in the scCO_2_ and the polymer phase. The impregnation of the polymer was continued for at least 2 h, and no significant changes in TEMPONE concentration were observed afterward. Next, chromatographic analysis of the supercritical phase was carried out. The medium from the autoclave was fed to the dosing loop through the 2 um inline filter (Waters, Milford, MA, USA), and all sampling parts were thermostated by a Termex M02 (Termex, Moscow, Russia) thermostat. The chromatographic column Luna C18-2 150 × 4.6 mm (Phenomenex, Torrance, CA, USA) was used. The composition of the mobile phase was 0.5% vol. methanol in CO_2_, the column temperature was 35 °C, and the backpressure was 120 bar. The quantitative analysis of the TEMPONE content in the supercritical phase was carried out according to a preliminarily obtained calibration curve. The calibration curve was obtained for up to ~23 mg loads of TEMPONE (corresponding to ~9.5 × 10^−4^ M) and remained linear. Hence, TEMPONE solutions in scCO_2_ were not saturated in all performed experiments. Thorough thermostating of all parts of the setup was found to be crucial to obtain a unique calibration curve for different temperatures. Although the content of TEMPONE in the supercritical phase was sufficient to calculate the distribution coefficient, we additionally measured the content of TEMPONE in PDLLA using EPR after the autoclave was depressurized. Both approaches led to, essentially, the same distribution coefficients. No signs of molecular aggregation of TEMPONE were found in the EPR spectra of the PDLLA samples.

### 2.3. Measurement of Polymer Impregnation Kinetics Using EPR Spectroscopy

**Figure 2:**
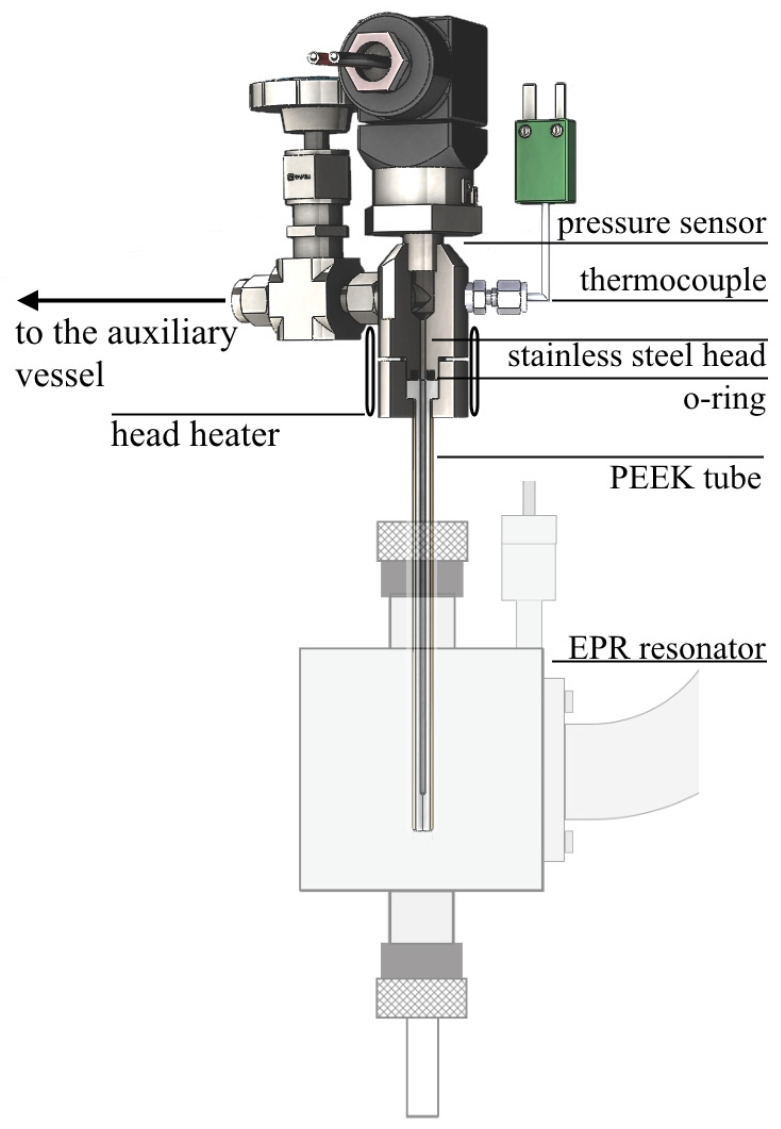
High-pressure setup for in situ EPR measurements.

The Electron paramagnetic resonance (EPR) measurements were carried out using a high-pressure system, previously described in detail [51,82,83]. The system consists of a custom-made polyetheretherketone (PEEK brand ZX-324, ‘Wolf-Kunststoff-Gleitlager GmbH’, Kerpen, Germany) tube (d_inner_ = 1.6 mm) with a stainless steel head (V_inner_ = 1.6 cm^3^), connected by a stainless steel capillary (inner diameter 0.8 mm) and high-pressure valves (‘Hy-Lok’, Busan, Republic of Korea) to an auxiliary vessel (V_inner_ = 49.2 cm^3^) (Figure 2). The head is mounted on the standard resonator of an X-band EPR spectrometer using a custom-made plastic holder allowing for a highly reproducible positioning of the sample in the resonator. The head and the auxiliary vessel were equipped with heating elements and PID controllers, while the tube temperature was maintained at the desired temperature by the standard temperature control system of the EPR spectrometer.

A weighted PDLLA sample (1–10 mg) either was used as a powder, or was bulk-cast in a glass capillary (d_inner_ = 1.1 mm), and was placed in the PEEK tube. A total of 50 μL of 0.2 M solution of TEMPONE in ethanol was added to the auxiliary vessel. The system was then evacuated, filled with CO_2_ at ~1 MPa, and evacuated again. The procedure was repeated several times to remove traces of oxygen and ethanol. Then, the valve between the auxiliary vessel and the tube was sealed, and the auxiliary vessel was heated to the desired temperature (313–333 K) and filled with CO_2_ (8.1–10.0 MPa); the pressures were taken from the NIST Chemistry WebBook database [84,85] so that the density of scCO_2_ remained constant (289.9 kg/m^3^). The auxiliary vessel was equilibrated for at least 30 min to ensure complete dissolution of TEMPONE in scCO_2_. Then, the system was transferred to the EPR spectrometer, the PEEK tube was equilibrated at the desired temperature, and the TEMPONE/scCO_2_ solution was let into the PEEK tube from the auxiliary vessel. TEMPONE was left to diffuse into PDLLA from scCO_2_ for 5 h, EPR spectra were recorded to obtain the number of TEMPONE radicals in the polymer during PDLLA impregnation.

EPR spectra were recorded using the Bruker EMX-500 spectrometer at temperatures of 313–333 K (set by a nitrogen flow and controlled with accuracy ± 1 K). The microwave radiation power was kept at 0.63 mW to avoid saturation, and the modulation amplitude was 0.5 G. The absolute number of paramagnetic particles was calculated by double integration of the EPR spectra. The resonator sensitivity profile over the tube was measured separately and accounted for. The concentration of TEMPONE in scCO_2_ (1.1 × 10^−4^ M) was determined from a calibration curve that was obtained in the same tube using standard solutions of TEMPONE in toluene.

PDLLA swells in scCO_2_ and forms a homogeneous polymer column at the bottom of the tube. Further, we can assume that the diffusion of the CO_2_ in PDLLA and the swelling of PDLLA are orders of magnitude faster than TEMPONE diffusion in swollen PDLLA [86,87,88], and we can suppose the areas achieved by the probe to be completely swollen. The diffusion of TEMPONE in PDLLA hence could be described as non-stationary one-component diffusion into a plane sheet from a medium with constant solute concentration. Mass balance of TEMPONE is, hence, described by [89]:

(1) $$N_{t}=N_{inf}\left(1-\frac{8}{\pi^{2}}\overset{\infty }{\underset{n=0}{\sum }}\frac{1}{{\left(2n+1\right)}^{2}}e^{\frac{-D{\left(2n+1\right)}^{2}\pi^{2}t}{4l^{2}}}\right)$$

where *N_t_* is the number of TEMPONE radicals in the plane sheet at the moment t, *N_inf_* is the number of TEMPONE radicals in the saturated with the spin probe plane sheet, *D* is the diffusion coefficient of the TEMPONE in swollen PDLLA, and l is the thickness of the plane sheet, which is the height of the swollen polymer column in our case. For relatively small times, the t Equation (1) is reduced to [89]:

(2) $$N_{t}=2N_{inf}\sqrt{\frac{Dt}{\pi l^{2}}}$$

We will rewrite Equation (2) in the form:

(3) $$\frac{N_{t}l\sqrt{\pi }}{2N_{inf}}=\sqrt{D}\sqrt{t}$$

The number of TEMPONE radicals at saturation limit was calculated using distribution coefficients as follows:

(4) $$N_{inf}=KC_{SCF}V_{PDLLA}N_{A}$$

where *K* is the distribution coefficient of TEMPONE between polymer and scCO_2_, *C_SCF_* is the concentration of the spin probe in scCO_2_, *V_PDLLA_* is the volume of the swollen polymer, and *N_A_* is the Avogadro constant. Diffusion coefficient *D* was then extracted from experimental TEMPONE uptake kinetics using the least-squares fitting with Equation (3).

### 2.4. Measurement of Concentration Broadening of EPR Spectra

We placed the samples of PDLLA uniformly impregnated with the TEMPONE spin probe (produced as described in Section 2.2) in the TEMPONE/scCO_2_ solution in the PEEK tube and obtained broadened EPR spectra of TEMPONE solutions in swollen PDLLA with concentrations of 8.9 × 10^−2^ M (1.5 wt%) and 1.6 × 10^−1^ M (2.9 wt%). The broadening is given by:

(5) $$∆B_{pp}=B_{pp}\left(∆C+C_{0}\right)-B_{pp}\left(C_{0}\right)$$

with *B_pp_*(*C*_0_) being the peak-to-peak linewidth of the EPR spectra of the infinitely diluted solution. There are two sources of concentration broadening: one is the dipole–dipole interaction, with the spins of the neighboring radicals, and the other is the Heisenberg spin exchange, due to the collisions of radicals. To the best of our knowledge, there is no analytical solution to the problem of distinguishing these two contributions in common use. The procedure to split the broadening into individual contributions proposed by Freed et al. [90] is not applicable here, due to the significant change of the PDLLA/scCO_2_ system properties with temperature and pressure [87]. However, it is known that the dipole–dipole contribution is averaged out at elevated temperatures, given the rotational correlation time is small enough [50,91]. Hence, we attributed the whole concentration broadening to the exchange interaction. Next, the spin-exchange rate constant is given by:

(6) $$k_{e}=\frac{\sqrt{3}\left|\gamma_{e}\right|∆B_{exchange}}{2\left(1-p\right)∆C}$$

where |*γ_e_*| is the gyromagnetic ratio, and *p* is the fractional degeneracy of the spectral transition (1/3 for ^14^N nuclei). On the other hand, *k_e_* can be expressed with diffusion coefficient D using the Einstein–Smoluchowski equation:

(7) $$k_{e}=16f\pi rD$$

where *r* is the radius of the paramagnetic particle, and *ƒ* is the steric factor. Here we stick to the values of *r* and *ƒ* derived by Freed et al. [90] (r = 6.4Å, ƒ = 0.678), of which a discussion can be found elsewhere [50]. EPR spectra of TEMPONE in PDLLA at 313–333 K were simulated using EasySpin [92]. The rotational motion of the TEMPONE molecules in swollen PDLLA was accounted for by a single rotational correlation time (τ_corr_) for each temperature.

## 3. Results and Discussion

### 3.1. TEMPONE Uptake Measurement

**Figure 3:**
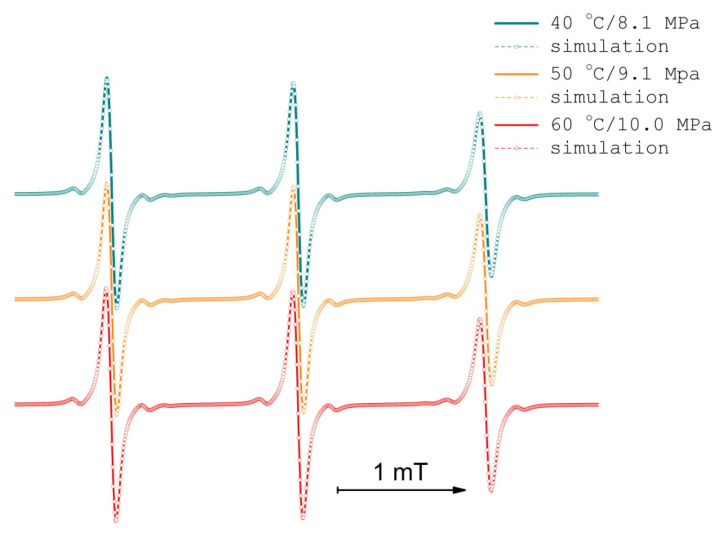
EPR spectra of TEMPONE in PDLLA swollen in scCO_2_ after impregnation during 5 h at scCO_2_ density of 289.9 kg/m^3^, isochoric conditions.

The TEMPONE nitroxide radical is readily absorbed by PDLLA upon exposure of the polymer to the solution of the nitroxide radical in scCO_2_. The manifestation of this process is the appearance of the EPR spectra with relatively narrow components (0.08 mT). The EPR spectra of TEMPONE in PDLLA swollen in scCO_2_ (PDLLA/scCO_2_) at 40–60 °C (Figure 3) are typical EPR spectra of nitroxide radicals in a viscous solution. The local mobility of the spin probe is readily available in the form of the rotational correlation time (τ_corr_, Table 1). The τ_corr_ of TEMPONE in PDLLA/scCO_2_ is larger than the τ_corr_ of TEMPONE in scCO_2_ (which is lower than the detectable minimum), resembles that of TEMPONE in water-glycerol mixtures, and is smaller than that of TEMPONE in pure glycerol solutions [93] (smaller τ_corr_ means higher local mobility). This means that microviscosity in glycerol > microviscosity in scCO_2_-swollen PDLLA = 1:1 water-glycerol mixture > microviscosity in scCO_2_. TEMPONE ^14^N hyperfine coupling (HFC) in swollen PDLLA (14.7 G) is slightly larger than TEMPONE ^14^N HFC in scCO_2_ (14.4 G), indicating a slightly higher local polarity in the swollen polymer than in the supercritical medium.

**Figure 4:**
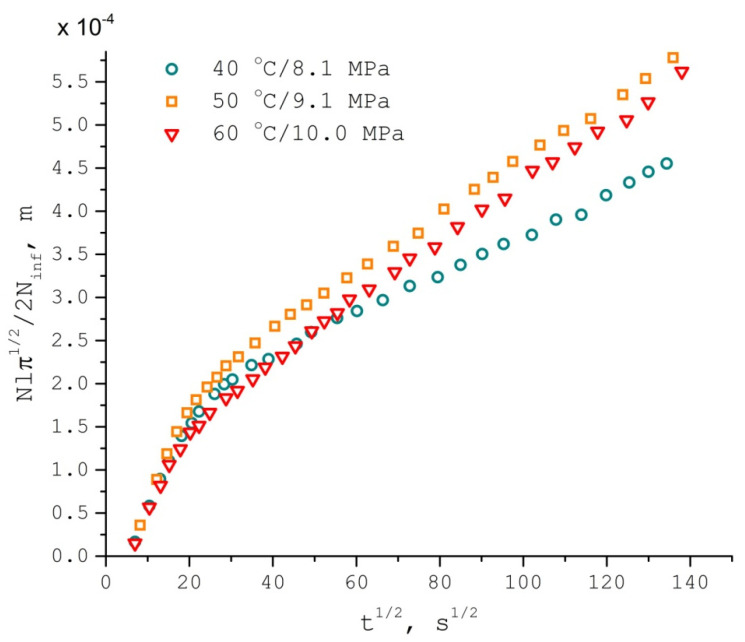
TEMPONE nitroxide radical normalized uptake by PDLLA swollen in scCO_2_ (density = 289.9 kg/m^3^, isochoric conditions) against the square root of time.

The amount of the spin probe in the polymer responsible for the narrow EPR spectra started to grow linearly with respect to t^1/2^ after approximately 15 min from the beginning of the experiment (Figure 4). This type of time dependence is typical for Fickian diffusion. The only values needed to bring the spin content of PDLLA during the impregnation to the form from Equation (3), that are not measured directly during the EPR experiment, were the distribution coefficients of TEMPONE between swollen polymer and supercritical medium. Hypothetically, those could also be measured during the EPR experiment given that the polymer reached a substantial degree of saturation with the spin probe. Though this would require either a significantly extended impregnation time or a significantly reduced polymer sample thickness. The former leads to an unreasonably long EPR experiment at elevated temperatures, and the latter requires a vanishingly small amount of polymer to be used, as EPR sample dimensions are severely restricted. Alternatively, outside the EPR cavity, one can use larger reactors and allow a substantial amount of PDLLA swollen by scCO_2_ to spread into a very thin layer and, thereby, reduce the time needed to perform the experiment. We used supercritical chromatography to analyze the TEMPONE content of the supercritical medium after the polymer was saturated with the spin probe. The measured distribution coefficients (K) are given in Table 1. The corresponding TEMPONE translational diffusion coefficients in the swollen PDLLA obtained from nitroxide uptake time dependence (D_uptake_) are also given in Table 1. Notably, diffusion coefficient values at 50 °C and 60 °C are similar. We assume that there are two opposing trends: on the one hand, the molecular mobility increases as the temperature rises; on the other hand, the mass fraction of a solvent in a polymer might decrease in isochoric conditions, resulting in a more viscous polymer medium. To support this assumption, we used the Sanchez–Lacombe equation of state (SLEOS) [94,95,96,97] given by:

(8) $${\tilde{\rho }}^{2}+\tilde{P}+\tilde{T}\left[\ln\left(1-\tilde{\rho }\right)+\left(1-\frac{1}{r}\right)\tilde{\rho }\right]=0$$

where reduced density, pressure, temperature, and the number of lattice sites occupied by a molecule are given by:

(9) $$\tilde{\rho }=\;\frac{\rho }{\rho^{*}},\;\tilde{P}=\frac{P}{P^{*}},\;\tilde{T}=\frac{T}{T^{*}},\;r=\frac{M}{\rho^{*}v^{*}}$$

where ρ*, P*, T*, and v* are characteristic density, pressure, temperature, and volume. M is the molecular weight. If applied to a binary system, the mixing rules for the characteristic parameters can be found elsewhere [96,97]. To estimate CO_2_ mass fraction in swollen PDLLA under the conditions used in the present paper, the PDLLA parameters of the SLEOS equation are taken from Liu and Tomasko [86] (Table 2). The calculated solvent mass fraction sharply decreases from 26 wt% at 40 °C to 19 wt% at 60 °C, which is why the diffusion coefficient values are not changed significantly.

**Figure 5:**
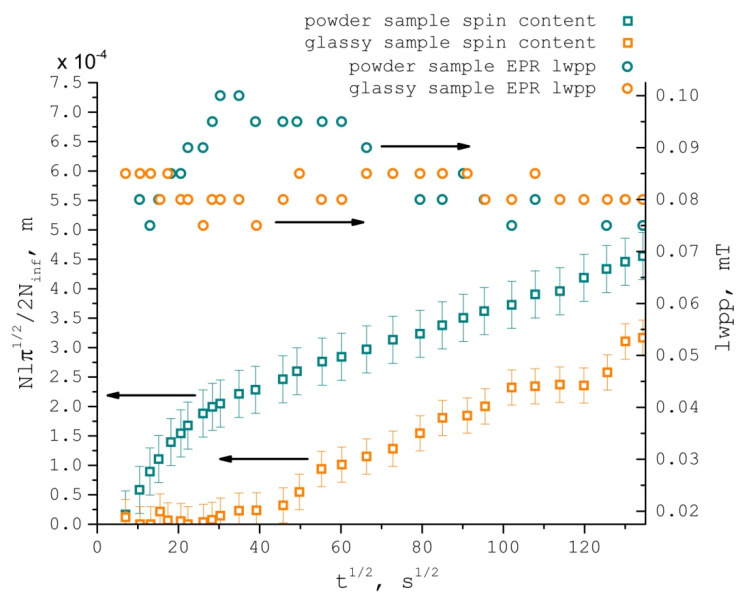
TEMPONE nitroxide radical normalized uptake by PDLLA swollen in scCO_2_ (density = 289.9 kg/m^3^) against the square root of time at 40 °C and 8.1 MPa and TEMPONE EPR spectra line widths in powder and glassy PDLLA samples.

The first segment of the TEMPONE uptake curves is essentially nonlinear in Fickian coordinates (Figure 4). We attribute this deviation to sorption and convective transport of the spin probe within the flow of CO_2_ during the initial swelling of the polymer. Indeed, there is a dramatic difference between TEMPONE uptake curves for different initial forms of the PDLLA samples (Figure 5). The powder PDLLA sample rapidly accumulates TEMPONE during the initial stage of polymer swelling, and the local concentration of the nitroxide radical in the polymer noticeably grows, as illustrated by the increase of the EPR linewidth, which is not typical for Fickian diffusion. On the other hand, the bulk glassy PDLLA sample shows very little uptake of TEMPONE during polymer swelling, with no local concentration increase. After PDLLA swelled and liquefied rapidly [86,87,88], the slopes of the uptake curves became equal for both types of polymer samples.

### 3.2. TEMPONE EPR Concentration Broadening Measurement

**Figure 6:**
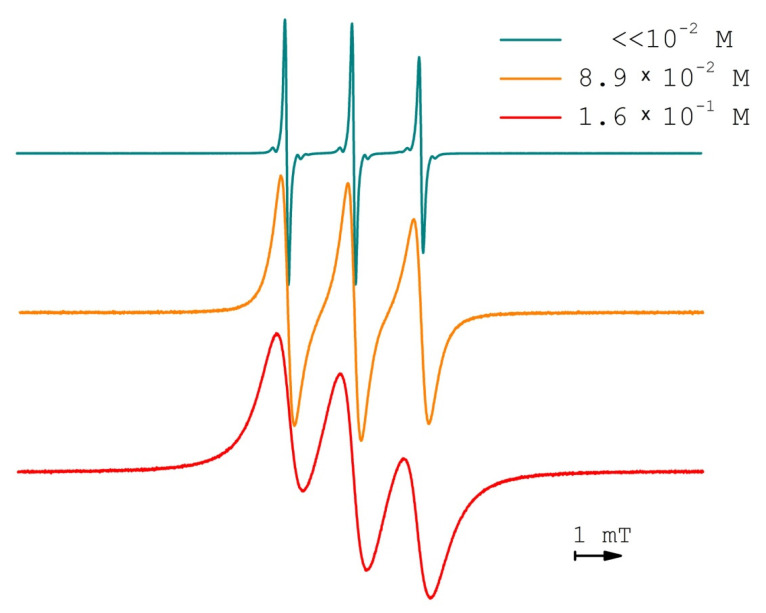
EPR spectra of TEMPONE in PDLLA swollen in scCO_2_ at 60 °C and 10.0 MPa (scCO_2_ density of 289.9 kg/m^3^).

The peak-to-peak linewidth (B_pp_) of EPR spectra components of the TEMPONE spin probe during PDLLA impregnation experiments did not depend on conditions and equaled to 0.08 mT (Figure 3). The concentration of TEMPONE in PDLLA during impregnation was always, on average, lower than 10^−2^ M. At higher concentrations, B_pp_ grows almost linearly, with respect to the concentration, due to collisions of nitroxide radicals leading to the spin-exchange reaction [30] and possibly dipole–dipole interaction (Figure 6). Relying on quite low rotational correlation times (Table 1) and, hence, high local mobility, the whole concentration broadening of the EPR spectra was supposed to originate from the spin exchange reaction while dipole–dipole interaction contribution was supposed to average out. The possibility of the additional plasticization of PDLLA by higher concentrations of TEMPONE was not taken into account.

**Figure 7:**
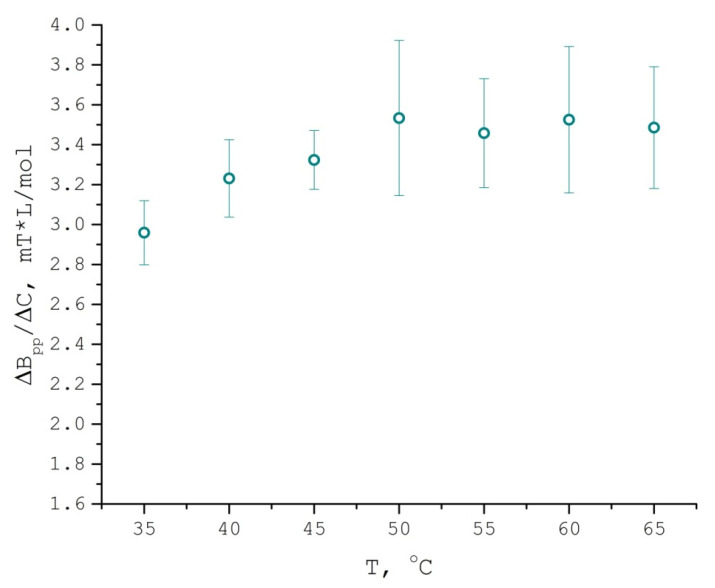
Concentration broadening (divided by concentration) of TEMPONE in PDLLA/scCO_2_ EPR spectra as a function of temperature (scCO_2_ density = 289.9 kg/m^3^, isochoric conditions).

Δ*B_pp_*/Δ*C* (Equation (5)) temperature dependence (Figure 7) exhibits the same trend as those observed in D_uptake_ and τ_corr_: spin probe rotational and translational mobility grows from 35 °C up to 50 °C, while from 50 °C to 65 °C it is maintained at the same level. This behavior does not fit the simple Arrhenius trend and is most likely due to a significant decrease of PDLLA swelling with temperature in accordance with our Sanchez–Lacombe equation of state calculations.

Combining Equations (6) and (7), we calculated the translational diffusion coefficients of TEMPONE in PDLLA/scCO_2_ from the Δ*B_pp_*/Δ*C* data. The obtained values (D_lwpp_, Table 1, where lwpp stands for peak-to-peak linewidth) are in agreement with D_uptake;_ hence, the impact of recollisions [99] does not seem to lead to overestimation of the translational diffusion rate. Furthermore, swollen PDLLA may be supposed to be rather homogenous on the macro- (> 1 μm path) and micro-scales (< 1 nm path), with no high-diffusivity paths and large pores, otherwise one would expect the diffusivity measured by line broadening to be higher than the diffusivity measured by solute uptake fitting. The absence of a mesoporous structure in the swollen PDLLA further follows from the absence of concentration dependence of diffusivities measured by spectrum line broadening [50]. In general transport properties of the swollen in scCO_2_ PDLLA resemble those of a viscous homogeneous liquid.

Even though prior knowledge of the distribution coefficients of TEMPONE between PDLLA and scCO_2_ is unnecessary to perform spectrum line broadening measurements, it is still desirable. Equilibration of the solute concentrations in supercritical medium and swollen polymer prevents the solute diffusion from the polymer, and hence guarantees uniform solute distribution with constant concentration over time, increasing thereby measurement accuracy. We should also note, that recent development by Salikhov et al. of a single spectrum-based exchange interaction rate measurement [100] can further facilitate estimation of the translational diffusion rate in similar systems and allow to eliminate the influence of the dipole–dipole interaction.

## 4. Conclusions

We applied established electron paramagnetic resonance (EPR) techniques and a relatively straightforward custom-built high-pressure setup, in conjunction with standard EPR equipment, to show the possibility of evaluating the transport properties of a polymer medium swollen in a supercritical fluid. Continuous-wave EPR allowed us to measure translational diffusion coefficients (D_tr_) of a TEMPONE spin probe in PDLLA polymer swollen in supercritical CO_2_ using two approaches. The first approach measures macroscopic diffusivity. It exploits the ability of EPR spectroscopy to measure the total content of the spin probe in the sample, which is then fitted to the well-known solution of Fick’s equation for diffusion in a plane sheet from a medium with constant concentration. The second approach measures microscopic diffusivity (<10 nm). It is based on the concentration-dependent broadening of the EPR spectra of a spin probe in the swollen polymer sample. Both methods yield similar translational diffusion coefficients and similar temperature dependencies of D_tr_ (in the range 5−10 × 10^−12^ m^2^/s). The absence of the concentration dependence of the measured D_tr_ leads to a conclusion that swollen PDLLA lacks mesoporous or an otherwise inhomogeneous structure. On the other hand, the TEMPONE spin probe in the swollen PDLLA exhibits higher rotational mobility than is expected based on its D_tr_. Furthermore, we were able to see the difference between the bulk sample and the powder sample impregnations: the former needs time to swell, and hence the start of the diffusion of the solute is retarded, while the latter readily gained a large amount of the solute presumably due to the convective transport mechanism during the polymer swelling. High-pressure EPR was found, hence, to reveal many interesting features of the transport processes in a swollen polymer under supercritical conditions. We should emphasize that the spin-probe high-pressure EPR technique is applicable to a wider range of objects than just swollen polymers. For example, protocols used to study the transport properties of porous materials, such as zeolites or aerogels, using conventional EPR [50,101] are easily transferred to high-pressure EPR.

## Figures and Tables

**Table 1:** Distribution coefficients (K) of TEMPONE between swollen PDLLA and scCO_2_ (density = 289.9 kg/m^3^); translational diffusion coefficients of TEMPONE nitroxide radical in PDLLA/scCO_2_ calculated from the uptake of TEMPONE by PDLLA time dependence (D_uptake_) and from concentration broadening of the EPR spectra of TEMPONE in PDLLA/scCO_2_ (D_lwpp_); rotational correlation times (τ_corr_) and ^14^N hyperfine coupling constant (a_iso_) of TEMPONE in PDLLA/scCO_2_.

T °C	K	D_uptake_, m^2^/s	D_lwpp_, m^2^/s	τ_corr_, s	a_iso_, G
40	520 ± 50	5 ± 2 × 10^−12^	7 ± 1 × 10^−12^	6.4 × 10^−11^	14.7
50	380 ± 80	10 ± 3 × 10^−12^	8 ± 1 × 10^−12^	5.7 × 10^−11^	
60	280 ± 50	10 ± 2 × 10^−12^	8 ± 1 × 10^−12^	5.5 × 10^−11^	

**Table 2:** PDLLA and CO_2_ SLEOS characteristic parameters.

Substance	T* (K)	P* (MPa)	p* (kg/m^3^)
PDLLA [86]	644.64	516.72	1331.3
CO_2_ [98]	269.5	720.3	1580

## Data Availability

The data presented in this study are available on request from the corresponding author.
